# Muscle Performance Changes with Age in Active Women

**DOI:** 10.3390/ijerph18094477

**Published:** 2021-04-23

**Authors:** Ryan M. Miller, Eduardo D. S. Freitas, Aaron D. Heishman, Keldon M. Peak, Samuel R. Buchanan, J. Mikhail Kellawan, Hugo M. Pereira, Debra A. Bemben, Michael G. Bemben

**Affiliations:** 1 Department of Health and Exercise Science, University of Oklahoma, Norman, OK 73019, USA; eduardofreitas@ou.edu (E.D.S.F.); aaronheishman@ou.edu (A.D.H.); Keldon.peak@ou.edu (K.M.P.); Jeremy.M.Kellawan-1@ou.edu (J.M.K.); Hugo.Maxwell.Pereira-1@ou.edu (H.M.P.); dbemben@ou.edu (D.A.B.); mgbemben@ou.edu (M.G.B.); 2 Department of Health and Human Performance, University of Texas Rio Grande Valley, Edinburg, TX 78539, USA; samuel.buchanan01@utrgv.edu

**Keywords:** aging, dynamometry, muscle, strength, women’s health

## Abstract

The purpose of this study was to examine age-related differences in muscle performance in women divided into young (YW, 20–39 years, *n* = 29) middle-aged (MAW, 40–59 years, *n* = 33), and older (OW, ≥60 years, *n* = 40) age groups. Methods: Hand grip strength, vertical jump performance, and knee extensor (KE) strength (0 deg/s, 60 deg/s, and 240 deg/s), speed of movement (SoM; at 1 Nm, 20%, 40%, and 60% isometric strength), and endurance (30-repetition test at 60 degs/s and 240 deg/s) were assessed. Computed tomography-acquired muscle cross-sectional area (mCSA) was measured and included to determine specific strength (KE strength/mCSA). Results: Hand grip strength was similar across groups, while jump performance declined with age (YW and MAW > OW, *p* < 0.001). KE strength declined significantly with age (all conditions *p* < 0.01), while specific strength was similar across groups. SoM was significantly higher for YW and MAW compared to OW (both *p* < 0.01). An age × velocity interaction revealed YW KE endurance was similar between conditions, whereas MAW and OW displayed significantly better endurance during the 60 deg/s condition. OW displayed impaired KE endurance at 240 deg/s (vs. YW and MAW, *p* < 0.01) but improved at 60 deg/s (vs. YW, *p* < 0.01). Dynamic torque decline increased with age (YW < OW, *p* = 0.03) and was associated with intramuscular adipose tissue (r = 0.21, *p* = 0.04). Conclusions: Performance declines were most evident among OW, but few performance deficits had emerged in MAW. Interestingly, strength declines disappeared after normalizing to mCSA and endurance appears to be velocity-dependent.

## 1. Introduction

The simultaneous reduction in muscle performance and mass with aging (i.e., sarcopenia) is associated with numerous adverse health events [1,2]. Interestingly, aging induces considerable strength declines [2,3], whereas declines in velocity/speed of movement (SoM) are less affected [4,5], and changes in endurance may be task/velocity-dependent [6,7]. Further, age-related changes in specific strength (strength normalized to site-specific muscle mass) remain unclear as previous results display comparable or lower specific strength among older adults [8,9,10,11,12]. However, recent evidence suggests that age-related differences in specific strength for women may be contraction-dependent since specific strength calculated from isometric knee extension performance was similar between young and older cohorts but older women displayed significantly lower specific strength when calculated from dynamic knee extension performance [10]. Nevertheless, for older adults, low muscle performance, mass, and specific strength are associated with adverse health events, mortality and increased healthcare costs [13,14,15,16,17,18,19]. Such associations warrant better comprehension of age-related changes in muscle performance.

Regarding low skeletal muscle performance and mass, women appear to generate greater healthcare costs than men [17,20]. One factor causing this difference may be conflicting age-related trajectories of muscle performance. Isokinetic dynamometry is often used in clinical and research settings to quantify muscle performance. Often, testing is completed in the isometric and isokinetic modes. The former measures strength at a fixed angle, whereas the latter measures dynamic strength across a specific range of motion at a constant velocity. Interestingly, although men and women display similar age-related reductions in lower extremity isometric strength, women display declines in dynamic strength significantly earlier than men [21]. Considering the importance of dynamic strength for completing activities of daily living [15,22], this contrasting difference is critical for maintaining physical performance during aging. Importantly, the authors noted the few available data examining high-velocity dynamic contractions (>180 deg/s), which ultimately limited such comparison. In contrast to the abovementioned contraction modes, isotonic contractions are performed with a predetermined load where SoM (deg/s) can be acquired and previous research reveals SoM may represent the most vital characteristic related to functional decline [16,23]. These gaps provide impetus to further examine age-related changes in high-velocity dynamic contractions and SoM.

To the best of our knowledge, only one study has examined skeletal muscle performance (strength, SoM, and endurance) and mass in young, middle-aged, and older women, which presents a gap in the current literature. Charlier et al. [4] observed declines of ~30% for knee extension strength, 13% for knee extensor SoM, and 40% for muscular endurance between young (18–30 years) and older (60–70 years) women. Men were also examined but the observed declines in muscle performance for women surpassed those for men. However, muscle endurance was limited to one contraction velocity (180 deg/s), there was no measure of specific strength, nor was a relatively emerging measure, dynamic torque decline calculated. Dynamic torque decline, the percent torque decline from slow to fast isokinetic contraction velocities, is thought to reflect age-related qualitative changes in skeletal muscle [24,25]. Despite such proposition, to our knowledge, only one previous study has linked poor muscle quality (via echo intensity) to dynamic torque decline [24], however, no previous research has examined the associations between dynamic torque decline and muscle cross-sectional area or adipose tissue in young middle-aged and older women, presenting an additional gap in the literature.

To extend previous research, we examined age-related changes in muscle performance and composition in women divided into young (20–39 years), middle-aged (40–59 years), and older (≥60 years) age groups. Our primary aim was to examine the influence of age on muscle performance measured by hand grip strength, vertical jump performance, and dynamometric knee extensor muscle performance. Knee extensor muscle performance was quantified via isometric strength, isotonic SoM, isokinetic strength (at 60 and 240 deg/s), and isokinetic endurance (at 60 and 240 deg/s). Our secondary aims included the examination of age-related changes in (i) specific strength, (knee extension strength relative to muscle cross-sectional area) and (ii) dynamic torque decline; and to (iii) examine the associations between dynamic torque decline and muscle composition. We hypothesized: (i) overall, muscle performance would decline with age; (ii) specific strength would be similar across age groups for isometric strength but not for isokinetic strength; and (iii) dynamic torque decline would increase with age and be associated with muscle composition.

## 2. Materials and Methods

### 2.1. Participants

A convenience sample of 102 women were divided into young (YW; 20–39 years, *n* = 29, 29.6 ± 6.3 years), middle-aged (MAW; 40–59 years; *n* = 33, 50.6 ± 5.5 years), and older groups (OW; ≥60 years; *n* = 40, 69.3 ± 7.9 years). All YW and OW were pre- or postmenopausal, respectively, whereas 48% (16/33) of MAW were premenopausal. Participants were classified as moderately active [26] self-reporting an average of 4.5 ± 1.6 days of exercise per week (range 2–7 days; YW: 4.5 ± 1.7 days; MAW: 4.7 ± 1.4 days; and OW: 4.5 ± 1.7 days, *p* = 0.91) but exercise did not exceed 1hour/day. All participants completed two laboratory-devised questionnaires first detailing demographic information and second regarding menstrual status (current status and previous history). Additionally, participants completed two validated physical activity questionnaires [26,27]. These questionnaires were completed during the initial visit to verify inclusion and exclusion criteria. Specific exclusion criteria consisted of the following: (i) training for a competitive event; (ii) currently smoking; (iii) known underlying chronic diseases; (iv) recent musculoskeletal injury (≤12 months); (v) stature exceeding bioimaging scanning guidelines; and (vi) current or previous exogenous hormone use. Participants were provided details outlining the risks and benefits associated with participation and provided written informed consent prior to completing study related procedures. This study was approved by the University of Oklahoma Institutional Review Board (No. 9838).

### 2.2. Study Design

Participants completed four visits which included: Visit 1—written consent, blood pressure reading, questionnaires, and muscle performance familiarization; Visit 2—bioimaging assessments, then muscular performance testing; and Visits 3 and 4—repeated muscle performance testing for reliability analyses. Muscle performance familiarization trials during Visit 1 consisted of participants performing each muscle performance testing procedure beginning with submaximal attempts (~50% maximal effort) and progressed to completing two to three near maximal efforts. During familiarization, participants were provided verbal instructions and then completed each test under supervision to demonstrate competency. During the familiarization trials, participant orientation was recorded to ensure consistent participant orientation across trials. Visits 2–4 were each separated by 7–10 days and were completed at similar times (±1 h) within the same participant. Prior to each testing visit, participants were asked to consume a light snack and to refrain from exercising at least 48-h prior to the scheduled visit.

### 2.3. Bioimaging Assessments

Height and weight were measured using a wall-mounted stadiometer (Novel Products, Rockton, IL, USA) and digital electronic scale (Tanita Inc., Arlington Heights, IL, USA), respectively. Before scanning, hydration status was determined with a urine refractometer (VEE GEE CLX-1, Rose Scientific Ltd., Edmonton, AB, Canada) to determine if participants met acceptable hydration levels [28]. Dual energy X-ray absorptiometry (DXA, Lunar Prodigy, GE Healthcare, Madison, WI, USA) measured total body composition and were performed and analyzed by the same trained technician (enCORE software, v16 GE Healthcare, Madison, WI, USA). Peripheral quantitative computed tomography (pQCT, XCT3000, v6.00 Stratec Medizintechnik gmbH, Pforrzheim, Germany) measured muscle cross-sectional area (mCSA, cm^2^) and intramuscular adipose tissue (IMAT, %), at the participant’s right 40% femur site determined as the distance from the top of the greater trochanter to the end of the lateral condyle of the tibia. During scans, participants sat upright with their right leg extended through the pQCT gantry. Immediately following the scan, visual inspection determined whether excess movement was observed [29] and if so, the scan was repeated. Due to excessive movement, pQCT data was unavailable for one MAW. Specific strength was calculated as knee extensor torque divided by thigh mCSA (e.g., isometric strength/mCSA) as suggested previously [30]. Prior to each scanning visit, DXA and pQCT quality assurance procedures were performed using manufacturer provided calibration phantoms for both bioimaging devices. Coefficient of variation (%CV) values from our laboratory for DXA and pQCT fat and muscle parameters are 1.21–3.97% and 1.40–2.92%, respectively.

### 2.4. Hand Grip Strength and Jump Performance

Hand grip strength was measured using a Jamar dynamometer (Sammons Preston, Bolingbrook, IL, USA) in the seated position for both hands. Participant hand grip span was self-selected during the familiarization trials and was recorded and kept consistent across testing visits. When testing, participants were asked to squeeze the device as hard as possible for three seconds then 30 s rest was provided before completing the next trial in the alternate hand. Three trials were performed for each hand and the maximal value across all trials for either hand was included in the analyses. The intraclass correlations coefficient (ICC) for hand grip strength was ICC_2,1_ 0.94.

Vertical jump velocity and power were measured via Tendo FiTRODYNE (Tendo Machines, Trencin, Slovak Republic). Participants were instructed to descend to a self-selected depth and then jump as high as possible with an unrestricted arm swing motion. Three trials were performed at each testing visit, with each trial separated by 60 s. Mean jump power (Watts) and jump velocity (m/s) values across visits 3 and 4 were included in the analyses. Relative jump power was calculated as jump power divided by body weight (Watts/kg). Test–retest reproducibility across participants for jump power and jump velocity were ICC_2,k_: 0.85 and 0.91, respectively.

### 2.5. Dynamometric Performance Assessments

Muscle performance of the right knee extensors was measured via isometric, isotonic, and isokinetic dynamometry (Biodex Systems 3, Shirley, NY, USA). Participant positioning and orientation followed manufacturer guidelines. The knee was fixed at 90° during isometric contractions, whereas the dynamic contractions had a range of motion from 90–160°. Participants completed muscle performance testing in the following order: (1) isometric contractions; (2) isotonic contractions; and (3) isokinetic contractions at 60 deg/s then 240 deg/s. A five-minute rest was provided between contraction modes, while 10 min rest was provided between isokinetic contraction velocities. For isometric testing, three, five-second contractions were performed, each separated by 60 s, where mean torque in Newton meters (Nm) was derived. Isotonic testing was performed against four external loads consisting of 1Nm and 20%, 40%, and 60% of the participant’s isometric maximum. Participants performed three, maximal ballistic knee extension contractions against each load in ascending order. Each contraction was separated by 60 s and one-minute rest was provided between different external loads. Mean velocity (deg/s) was derived from isotonic contractions. Isokinetic testing consisted of 30 reciprocal knee extension–flexion contractions at 60 deg/s and 240 deg/s. Mean torque (Nm) derived from the first three knee extension repetitions was included in the current analyses and dynamic torque decline was calculated as the percent decline in torque from 60 deg/s to 240 deg/s (i.e., greater values represent greater percent decline from torque at 60 deg/s vs. 240 deg/s. Muscular endurance was calculated from the total amount of work performed during the first and last ten repetitions calculated as follows: (100 - [work from last 10 repetitions/work from first 10 repetitions) × 100] = % decrease). This value is calculated within the Biodex software and a greater value represents lower muscular endurance [31]. Mean values across Visits 3 and 4 (six total trials for isometric strength, isotonic SoM, and isokinetic strength, two for muscular endurance) were included in the current analyses. Based on the mean-rating, the absolute-agreement, two-way mixed-effects model revealed that all tests achieved at least moderate reliability (all ICC_2,k_: ≥0.74) [32].

### 2.6. Statistical Analyses

Statistical analyses were performed using R Studio 3.6.1 (R Foundation, Vienna, Austria) and values are displayed as mean ± SD. One-way analyses of covariance (ANCOVA) examined participant characteristics, hand grip strength, vertical jump performance, and dynamic torque decline adjusted for height. Next, several two-way (contraction condition × age) mixed factorial analyses of covariance (ANCOVA) compared dynamometric muscle performance across age groups with height as a covariate. When significant differences were observed (*p* < 0.05), post-hoc comparisons were performed using the Bonferroni correction to adjust for multiple comparisons. Pearson’s correlation and partial correlation coefficients (adjusted for age) examined associations between muscle composition (mCSA or IMAT) and dynamic torque decline. Prior to examining associations, dynamic torque decline and IMAT were log-transformed to approximate normal distributions.

## 3. Results

### 3.1. Participants

**Figure 1:**
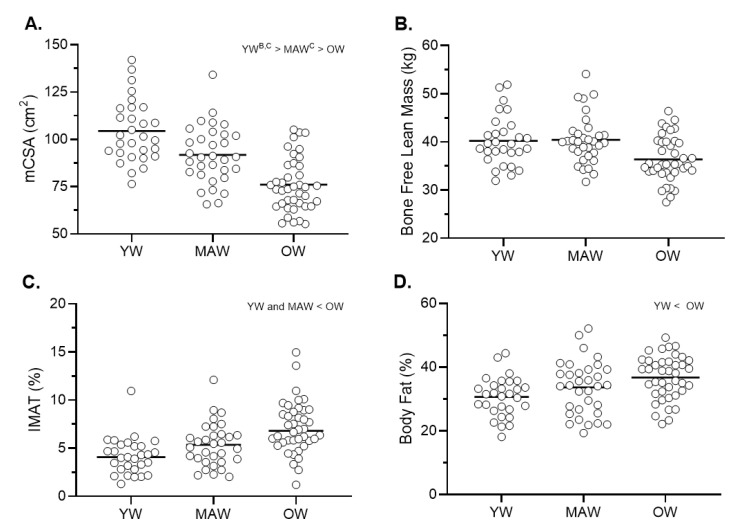
Body and Thigh Composition by Age Group. Notes: Panel (**A**)—Muscle cross-sectional area (mCSA); Panel (**B**)—bone free-lean mass; Panel (**C**)—Intramuscular adipose tissue (IMAT, %); Panel (**D**)—Total body fat (%). Letters reflect ANCOVA post-hoc significant differences (*p* < 0.0167) from respective group (a—young women; b—middle-aged women; c—older women).

Table 1 presents the participant characteristics. Groups differed significantly in age (YW < MAW < OW; all *p* < 0.001). Height displayed a significant effect for age (*p* = 0.004), which indicated that MAW were significantly taller than OW (*p* < 0.01) but not YW. Figure 1 presents individual body and thigh composition parameters. Bone free-lean mass and percent fat displayed significant group effects (both *p* ≤ 0.004). More specifically, YW and MAW displayed significantly greater bone free-lean mass than OW (both *p* < 0.01), while the fat percentage was significantly higher in OW compared to YW (*p* < 0.01). Significant group effects were observed for each of the midthigh composition parameters (all *p* < 0.001). Muscle cross-sectional area was significantly different across all groups (YW > MAW > OW; all *p* < 0.01), whereas thigh IMAT was significantly greater in the OW compared to both YW and MAW (both *p* < 0.01).

### 3.2. Hand Grip Strength and Vertical Jump Performance

There was no difference in hand grip strength across age groups (YW: 30.3 ± 4.8 kg; MAW: 30.1 ± 4.4 kg; and OW: 27.2 ± 4.3 kg; all *p* > 0.03). Vertical jump performance displayed significant group effects (Table 1, all *p* < 0.001). Post-hoc analyses revealed YW and MAW displayed significantly greater absolute and relative jump power and jump velocity compared to OW (all *p* < 0.01), while similar performance was noted between YW and MAW (all *p* > 0.09).

### 3.3. Dynamometric Muscle Performance

**Figure 2:**
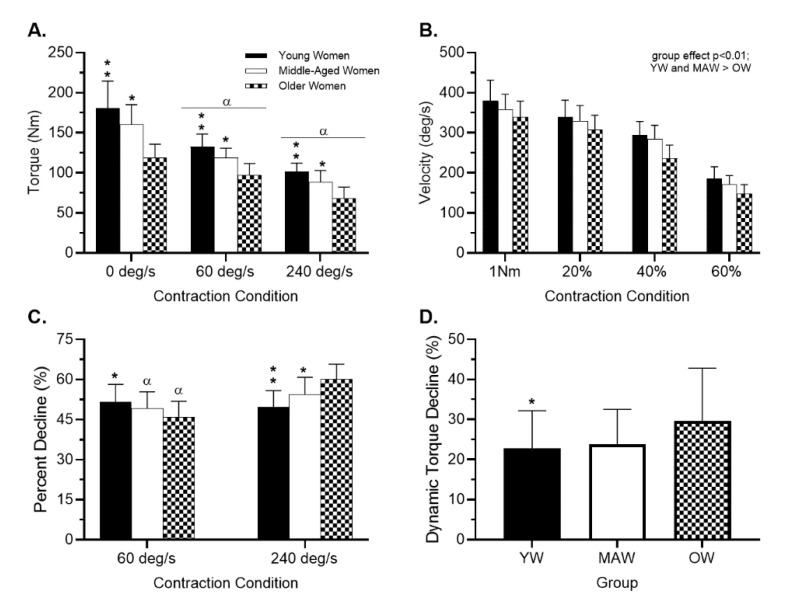
Age-associated Changes in Muscle Performance. Notes: Panel (**A**)—isometric and dynamic strength; Panel (**B**)—isotonic contraction velocity; Panel (**C**)—isokinetic muscular endurance showed as percent decline in torque (greater endurance is represented by lower values); Panel (**D**)—dynamic torque decline (greater values represent larger reduction in torque when isokinetic speed is increased from 60 to 240 deg/s). Panels (**A**–**C**): Bars represent young women (filled); middle-aged women (open); and older women (hatched). Panel (**D**): YW—young women; MAW—middle-aged women; OW: older women. Values are presented as unadjusted mean ± standard deviation. Significance derived from ANCOVA analyses with height inserted as covariate. Single asterisk denotes significant difference from older women; stacked asterisk denotes significant difference from middle-aged and older women; alpha represents condition effect.

Figure 2 Panels A–D and Supplementary Table S1 displays knee extensor dynamometric performance. Absolute torque displayed a significant age × condition interaction (*p* < 0.001, $\eta_{p}^{2}$: 0.19). Post-hoc analyses revealed that all three groups displayed significantly different (all *p* < 0.01) absolute isometric and isokinetic torque (both 60 deg/s and 240 deg/s) compared to the alternative groups (YW > MAW > OW, all *p* < 0.01). Additionally, all three groups displayed significantly different absolute torque across conditions (0 deg/s > 60 deg/s > 240 deg/s, all *p* < 0.01). Knee extensor SoM did not reveal a significant group × condition interaction (*p* = 0.07, $\eta_{p}^{2}:$ 0.04) or condition effect (*p* = 0.39, $\eta_{p}^{2}:$ 0.01), but displayed a significant group effect (*p* < 0.01 $\eta_{p}^{2}$: 0.30). Collapsed by group, post-hoc analyses revealed YW and MAW displayed significantly greater SoM than OW (both *p* < 0.01), while YW and MAW displayed similar SoM (*p* = 0.09).

Isokinetic muscle endurance displayed a significant group × velocity interaction (*p* < 0.001, $\eta_{p}^{2}:$ 0.40). During the 60 deg/s YW displayed significantly greater torque decline compared to OW (*p* < 0.01) but MAW were similar to both YW and OW (*p* = 0.07 and *p* = 0.08, respectively). At 240 deg/s, all three groups displayed significantly different declines compared to the alternative groups (all *p* < 0.01). Within groups, YW displayed similar percent decline between conditions (*p* = 0.20), whereas MAW and OW displayed significantly greater percent decline during the 240 deg/s condition (both *p* < 0.01).

Specific strength displayed a significant group × velocity interaction (*p* = 0.05, $\eta_{p}^{2}:$ 0.06). Post-hoc analyses did not detect significant group differences for specific strength at 0 deg/s (YW: 1.7 ± 0.3 Nm/cm^2^; MAW: 1.8 ± 0.4 Nm/cm^2^; OW: 1.6 ± 0.4 Nm/cm^2^) 60 deg/s (YW: 1.3 ± 0.2 Nm/cm^2^; MAW: 1.3 ± 0.3 Nm/cm^2^; OW: 1.3 ± 0.3 Nm/cm^2^) or 240 deg/s (YW: 1.0 ± 0.2 Nm/cm^2^; MAW: 1.0 ± 0.2 Nm/cm^2^; OW: 0.9 ± 0.3 Nm/cm^2^). Last, for all groups specific strength was significantly different across conditions (1.7 ± 0.4 Nm/cm^2^, 1.3 ± 0.3 Nm/cm^2^, 1.0 ± 0.2 Nm/cm^2^ for 0, 60, and 240 deg/s, respectively, all *p* < 0.01).

Significant group differences were detected for dynamic torque decline (YW: 22.9 ± 9.4%; MAW: 25.2 ± 12.4%; and OW: 29.5 ± 13.4%, *p* = 0.03). Post-hoc analyses revealed YW displayed significantly lower dynamic torque decline compared to OW (*p* = 0.01), while MAW displayed similar dynamic torque declines to both YW and OW (*p* = 0.75 and *p* = 0.03, respectively). Dynamic torque decline was significantly associated with IMAT (*r* = 0.27, *p* = 0.01), whereas mCSA was not (*r* = −0.11, *p* = 0.26). After adjusting for age, the association between dynamic torque decline and IMAT remained significant (*r* = 0.20, *p* = 0.04).

## 4. Discussion

The purpose of the current study was to examine age-related differences in skeletal muscle performance, composition, and specific strength across the adult female lifespan. Our primary observations indicate: (i) lower extremity absolute strength decreased significantly with ascending age groups (YW > MAW > OW, all *p* < 0.01); (ii) jump performance and knee extension SoM were similar between YW and MAW, but was significantly lower in OW compared to YW and MAW (all *p* < 0.01); (iii) increasing age affects knee extensor muscular endurance, as shown in Figure 2C by the percentage decline in force, in a velocity-dependent manner; (iv) knee extensor specific strength was similar across age groups highlighting the importance of mCSA in torque production; and (v) dynamic torque decline in OW was significantly greater than YW (*p* < 0.01) and was associated with IMAT (*p* = 0.04).

Hand grip strength did not differ across the three groups. Based on normative values, hand grip strength for the YW and MAW were in the 75th percentile compared to the 90th percentile for OW [33]. Considering the prognostic value of hand grip strength [34,35], the percentile differences may reflect current health status (e.g., healthier subset of OW compared to YW and MAW) and help explain the preserved hand grip strength with increasing age. Supporting previous work, vertical jump performance declined with age [36,37]. Jump performance declines were likely driven by a speed component (i.e., acceleration or SoM) since body mass was similar across groups. It is also possible the observed declines in knee extensor strength may also contribute. Interestingly, maintained jump performance in MAW may reflect maintained velocity which may have compensated for significant strength declines. Nevertheless, vertical jump tests provide unique clinical and practical relevance since the test provides a simultaneous and coordinated assessment of parameters dependent upon an individual’s body weight, which is also the same resistance that individuals encounter during activities of daily living. As such, recent observations from the Hertfordshire Cohort Study suggest that vertical jump parameters are more sensitive indicators of fall risks than traditional physical function tests (e.g., timed up and go, hand grip strength, etc.) [38].

Collectively, age-related strength declines of the lower extremity were similar across conditions (0 deg/s: 34%; 60 deg/s: 27%; 240 deg/s: 33%) but exceeded those for SoM (11–21%). These observations parallel previous results in women [4,5,39] and collectively suggest that strength rather than SoM displays greater declines with age in women. Such observations contain practical relevance that may help guide intervention optimization. For example, since muscular power (strength × SoM) is an integral component for successful aging [40,41], the larger declines in strength may represent a more modifiable performance attribute towards augmenting muscular power. These suggestions are bolstered by recent systematic review and meta-analytic work illustrating the positive relationship between load magnitude and bone response in postmenopausal women [42,43]. Therefore, prescribing high loads may not only augment muscular adaptations but may also provide an osteoprotective stimulus.

To date, few studies have examined muscle endurance across the lifespan with scarce data from women [4,44,45,46]. Our results highlight the influence of age and velocity on muscle endurance. Presumably, age-related changes in muscle fiber type [47] may provide an advantage for older adults during the 60 deg/s condition. In contrast, muscle endurance declined (greater percent decline) across increasing age groups during the 240 deg/s condition. Declines during the 240 deg/s condition may reflect metabolite accumulation, which can limit muscular performance through a variety of mechanisms, such as inhibiting crossbridge cycling, affecting calcium kinetics, and reducing force per crossbridge, among other consequences [48,49,50,51,52]. Collectively, these observations support previous notions that age-related changes in muscle endurance are influenced by contraction velocity [53,54,55] and that potential advantages during low-velocity condition may not be transferred to high-velocity tasks requiring muscle endurance.

Previous research posits dynamic torque declines may reflect age-related differences in skeletal muscle quality [24,25]. Supporting previous research, we observed significant declines in dynamic torque decline with age [24,25]. However, the current results extend previous findings since we included a middle-aged group, ultimately showing such deficits arise at older ages. Interestingly, Gerstner et al. [24] reported greater echo intensity (lower muscle quality) was associated with larger dynamic strength declines. Herein, we also report qualitative changes in skeletal muscle (increased IMAT) with age that were significantly associated with larger dynamic torque decline, ultimately supporting the aforementioned hypotheses. However, to our knowledge, we are the first to identify this association, thus additional research is needed to bolster this novel result. Further evidence for this association may encourage the development for lifestyle—or pharmacological-based intervention that targets IMAT as a therapeutic target towards improving physical function and muscle strength. Additionally, future research should examine whether dynamic torque declines reflect physical performance better than a single contraction (i.e., 0 deg/s or 60 deg/s), which contains pertinent prognostic value for identifying at-risk individuals.

The current observations suggest specific strength is not affected by age. Such results persisted across isometric and isokinetic (60 and 240 deg/s) conditions, suggesting that contraction type may not influence specific strength. Regarding isometric specific strength, our findings support previous observations among women [4,8,10,56,57,58], which lends support that mCSA is a primary contributor to muscle strength in women. However, in contrast to our results, although Charlier et al. [4] reported no age-related differences in isometric specific strength, increasing age resulted in significant declines in specific strength at 60 deg/s and 240 deg/s. Methodological differences, such as distinct approaches for determining specific strength presumably contribute to the different observations. Specifically, Charlier et al. [4] used skeletal muscle index (lean mass/height) to normalize strength whereas the current study used mCSA. Nevertheless, the ambiguity surrounding specific strength determination underscores the need to determine a standardized assessment [59]. However, previous research demonstrates that independent of muscle mass, muscle performance (i.e., strength, SoM, and endurance) is as capable as specific strength in conveying physical status [13,14,15,16,60,61,62]. Such observations ultimately challenge the clinical utility of specific strength.

Our study contained notable strengths, such as the inclusion of women, a traditionally underrepresented group of participants in biomedical research [63]. Further, numerous practical (i.e., hand grip strength and vertical jump) and laboratory-based (dynamometric performance, DXA, pQCT) muscle measures were assessed, in turn providing an extensive examination of age-related changes. Further, participants were generally healthy, recreationally active females, which may more effectively reflect trajectories of natural aging [64]. However, the current study contains limitations worth mentioning. First, these are cross-sectional observations from a relatively small number of healthy females, thus inferring causal relationships with age or similar patterns in alternative populations would be inappropriate. Given the sample size, it is possible age-related changes in specific strength went undetected due to its small effect. Second, we did not examine additional factors known to influence age-related changes in muscle performance and composition (e.g., hormones and dietary intake). Third, muscle performance tests were not randomized across participants, thus there is a chance that fatigue accumulation may influence the current results. Nevertheless, the noted limitations provide avenues for future research.

## 5. Conclusions

In summary, the current observations suggest that (i) strength declines exceed those for SoM; (ii) muscle endurance, but not specific strength, is velocity-dependent; and (iii) dynamic torque decline increases with age and is associated with IMAT. Collectively, these observations may provide insight towards optimizing current lifestyle interventions among OW where deficits became most evident. For example, interventions designed to maintain muscle composition (i.e., maximize mCSA retention and minimize IMAT accumulation) may help attenuate age-related declines in muscle performance. Further, it is possible that interventions designed to improve absolute torque production (i.e., strength) rather than SoM may confer greater benefit given the larger age-related declines differences between YW and OW. Importantly, further research is needed to support the novel observation regarding the association between dynamic torque decline and IMAT.

## Figures and Tables

**Table 1:** Participant Characteristics by Age Group.

Parameter	Young Women	Middle-Age Women	Older Women	Age Effect	^1^ Post-hoc
Participants (*n*)	29	33	40	–	–
Age (years)	29.6 ± 6.3	50.6 ± 5.5	69.3 ± 7.9	**<0.001**	YW < MAW < OW
Height (cm)	166.3 ± 6.7	166.6 ± 6.9	161.8 ± 6.7	**0.004**	MAW > OW
Weight (kg)	65.5 ± 7.9	69.3 ± 12.0	64.6 ± 10.4	0.140	Nc
Body mass index (kg/m^2^)	23.7 ± 3.1	25.0 ± 4.7	24.7 ± 3.6	0.392	Nc
Total Physical Activity	24.5 ± 30.6	24.9 ± 21.4	17.8 ± 19.2	0.366	Nc
Bone free-lean mass (kg)	40.2 ± 5.2	40.4 ± 5.0	36.4 ± 4.6	0.077	Nc
Fat Percent (%)	30.7 ± 6.2	33.6 ± 8.4	36.7 ± 6.9	**0.031**	YW < OW
Thigh mCSA (cm^2^)	104.5 ± 16.5	91.8 ± 15.2	76 ± 14.1	**<0.001**	YW > MAW > OW
Thigh IMAT (%)	4.2 ± 1.9	5.4 ± 2.3	7.2 ± 2.7	**<0.001**	YW and MAW < OW
Jump power (Watts)	1129.5 ± 215.4	1045.1 ± 159.1	751.0 ± 220.3	**<0.001**	YW and MAW > OW
Jump velocity (m/s)	1.3 ± 0.2	1.2 ± 0.1	1.0 ± 0.2	**<0.001**	YW and MAW > OW
Relative jump power (Watts/kg)	17.5 ± 4.2	15.7 ± 4.2	12.0 ± 4.4	**<0.001**	YW and MAW > OW

Abbreviations: cm—centimeters, kg—kilograms, kg/m^2^—kilograms per meters squared, m/s—meters per second, mCSA—muscle cross-sectional area, IMAT—intramuscular adipose tissue, YW—young women, MAW—middle-age women, OW—older women, NC—no post-hoc comparison. ^1^—Post-hoc presents significant pairwise comparisons at *p* < 0.0167 for body/thigh composition and jump performance. Bold values are significant.

## Supplementary Materials

The following are available online at https://www.mdpi.com/article/10.3390/ijerph18094477/s1, Table S1: Summary of Dynamometric Muscle Performance.
