# Supplementary material for: Muscle Performance Changes with Age in Active Women
Source: Int J Environ Res Public Health. 2021 Apr 23;18(9):4477. doi: 10.3390/ijerph18094477 (PMC8122865; doi:10.3390/ijerph18094477)
Supplement: Supplementary file 1 [file ijerph-18-04477-s001.zip › ijerph-1178345-supplementary.pdf]

Supplementary Table 1. Summary of dynamometric muscle performance.

	Age Group			ANOVA Results[1]		
Absolute Torque	YW	MAW	OW	Age × Condition	Age	Condition
0deg/s (Nm)	180.7 ± 33.8[B,C†]	161.1 ± 25.4[C†]	118.7 ± 17.1[†]	**<0.001** [0.18]	**<0.001** [0.61]	0.089 [0.03]
60deg/s (Nm)	132.9 ± 15.6[B,C†]	118.2 ± 9.9[C†]	97.1 ± 14.4[†]			
240deg/s (Nm)	101.6 ± 10.5[B,C†]	90.0 ± 12.1[C†]	67.8 ± 14.4[†]			
Speed of Movement	YW[‡]	MAW[‡]	OW	Age × Condition	Age	Condition
1Nm (deg/s)	379.4 ± 51.8	359.2 ± 35.8	339.1 ± 39.7	0.072 [0.04]	**<0.001** [0.30]	0.394 [0.01]
20% (deg/s)	339.6 ± 41.8	330.1 ± 37.2	307.7 ± 35.7			
40% (deg/s)	294.2 ± 34.2	281.8 ± 33.4	236.4 ± 33.0			
60% (deg/s)	186.1 ± 29.0	171.5 ± 22.1	147.6 ± 23.1			
Endurance	YW	MAW	OW	Age × Condition	Age	Condition
60deg/s (% decline)	51.7 ± 6.5[C]	48.7 ± 6.2[†]	45.8 ± 6.0[†]	**<0.001** [0.40]	0.152 [0.04]	0.290 [0.01]
240deg/s (% decline)	49.6 ± 6.2[B,C]	54.4 ± 6.7[C]	60.2 ± 5.5			
Specific Strength	YW	MAW	OW	Age × Condition	Age	Condition
0deg/s (Nm/cm^2)	1.74 ± 0.3[†]	1.79 ± 0.4[†]	1.63 ± 0.4[†]	**0.048** [0.06]	0.133 [0.04]	**0.028** [0.04]
60deg/s (Nm/cm^2)	1.28 ± 0.2[†]	1.32 ± 0.2[†]	1.32 ± 0.3[†]			
240deg/s (Nm/cm^2)	0.98 ± 0.2[†]	0.99 ± 0.2[†]	0.93 ± 0.2[†]			

Abbreviations: Nm- Newton meters; deg/s- degrees per second, YW- young women, MAW- middle-age women, OW- older women, NC- no post-hoc comparison, NS- not significant. Means displayed as unadjusted means ± SD. 1- results displayed as p-value [partial eta squared], B- significantly different from MAW, C- significantly different from OW, ‡- significantly different from OW, †-significant condition comparison.
